# Pan-Cancer Analysis Reveals FH as a Potential Prognostic and Immunological Biomarker in Lung Adenocarcinoma

**DOI:** 10.1155/2021/8554844

**Published:** 2021-10-26

**Authors:** Heng Zhang, Qiang Ju, Jing Ji, Yanjie Zhao

**Affiliations:** ^1^School of Public Health, Qingdao University, Qingdao, Shandong, China; ^2^Department of Blood Transfusion, The Affiliated Hospital of Qingdao University, Qingdao, Shandong, China

## Abstract

Fumarate hydratase (FH) is an important enzymatic component in the tricarboxylic acid cycle. Studies have reported that FH plays an important role in hereditary leiomyomatosis and renal cell cancer (HLRCC). However, the role of FH in human different cancers remains unknown. This study is aimed at analyzing the prognostic value of FH and demonstrating the correlation between FH expression and tumor immunity. Results showed that FH was mutated or copy number varied in 27 types of cancer. FH mRNA was abnormally upregulated across various cancers. Survival analysis suggested high expression of FH was associated with poor prognosis in many cancer types, including lung adenocarcinoma (LUAD). Additionally, FH expression was associated with immune infiltration, including B cells, CD4^+^ T cells, CD8^+^ T cells, neutrophils, macrophages, and dendritic cells, especially in liver hepatocellular carcinoma (LIHC), LUAD, and lung squamous cell carcinoma (LUSC). Moreover, FH expression showed a strong correlation with immune checkpoint markers in LUAD and testicular germ cell tumors (TGCT). These results indicate that FH is an immunotherapeutic target and a potential prognostic biomarker in LUAD.

## 1. Introduction

Cancer is the leading cause of death worldwide, and most existing therapies are low effective [1–3]. Pan-cancer analyses can help us to find common and different characteristics of human malignant tumors [4] and provide novel ideas for the clinical treatment of tumors [5], for example, applying pan-cancer analysis to reveal that immune infiltration influences radiotherapy outcomes [6] and to explore the association between matrisome genes and tumors [7]. In addition, pan-cancer analysis can be used to find valuable prognostic biomarkers [8–10]. Therefore, pan-cancer analysis is an important method for identifying new diagnostic biomarkers and developing more effective molecular targets for cancer treatment.

Fumarate hydratase (FH) is an enzymatic component of the tricarboxylic acid cycle catalyzing fumarate to malate [11]. A growing number of studies have shown that FH is involved in the occurrence and development of certain cancers. For instance, patients with FH gene mutations have a very high risk of hereditary leiomyomatosis and HLRCC [12]. And gastric cancer patients with high FH expression had a higher risk of death than those with low FH expression [13]. In addition, the loss of FH and the accumulation of fumarate elicit an epithelial-to-mesenchymal-transition (EMT) to promote cancer metastasis [14, 15]. However, the role of FH in pan-cancers needs further study.

The occurrence and development of cancer are closely related to the surrounding stroma. Immune cells play important roles in the occurrence and progression of tumors and are crucial parts of tumor stroma [16, 17]. Tumor-associated macrophages (TAMs) are important immune cells in the tumor microenvironment and play protumoral or antitumoral roles [18, 19]. Therefore, the study of tumor immune microenvironment can provide new clues for understanding the mechanism of tumor occurrence and development and has important value for the clinical treatment of tumors. However, the current research on the role of FH in tumor immunity is still limited.

In this study, we analyzed the expression of FH and evaluated its prognostic value in 33 cancer types. More importantly, we explored the relationship between FH expression and various tumor immunities. Our results provide new insights into the role of FH in tumors, suggesting that FH is related to the immune infiltration of a variety of tumors and is a potential prognostic biomarker, especially in lung adenocarcinoma (LUAD).

## 2. Materials and Methods

### 2.1. Pan-Cancer Analysis of Mutational Data of FH

The mutation and amplification levels of FH in human cancers were evaluated by cBioPortal database (http://www.cbioportal.org/, v3.6.20). By using TCGA database (https://gdc.cancer.gov/access-data/gdc-data-transfer-tool, v23.0), we obtained the mutation levels of five mismatch repair (MMR) genes (MLH1, MSH2, MSH6, PMS2, and EPCAM). The correlation between FH level and MMR gene mutation level was explored by the Pearson correlation analysis.

### 2.2. Patient Datasets and FH Expression Analysis

The data of the FH expression in tumor and normal tissues of 33 types of cancers were obtained from the Genotype Tissue Expression (GTEx) (https://gtexport.org/home/, v8) and The Cancer Genome Atlas (TCGA). Clinical annotations and RNA sequencing data of 33 cancer types (ACC: adrenocortical carcinoma; BLCA: bladder urothelial carcinoma; BRCA: breast invasive carcinoma; CESC: cervical squamous cell carcinoma; CHOL: cholangiocarcinoma; COAD: colon adenocarcinoma; DLBC: lymphoid neoplasm diffuse large B cell lymphoma; ESCA: esophageal carcinoma; GBM: glioblastoma multiforme; LGG: brain lower grade glioma; HNSC: head and neck squamous cell carcinoma; KICH: kidney chromophobe; KIRC: kidney renal clear cell carcinoma; KIRP: kidney renal papillary cell carcinoma; LAML: acute myeloid leukemia; LIHC: liver hepatocellular carcinoma; LUAD: lung adenocarcinoma; LUSC: lung squamous cell carcinoma; MESO: mesothelioma; OV: ovarian serous cystadenocarcinoma; PAAD: pancreatic adenocarcinoma; PCPG: pheochromocytoma and paraganglioma; PRAD: prostate adenocarcinoma; READ: rectum adenocarcinoma; SARC: sarcoma; SKCM: skin cutaneous melanoma; STAD: stomach adenocarcinoma; TGCT: testicular germ cell tumors; THCA: thyroid carcinoma; THYM: thymoma; UCEC: uterine corpus endometrial carcinoma; UCS: uterine carcinosarcoma; and UVM: uveal melanoma) were obtained from TCGA. All data were normalized as previously described [20, 21].

### 2.3. Cell Culture and Reagents

BEAS-2B, 16HBE, A549, and H460 were obtained from the American Type Culture Collection (ATCC, Manassas, USA). 16HBE, BEAS-2B, and H460 were cultured in MEM medium (HyClone, Utah, USA) with 10% fetal bovine serum (FBS, Gibco, Amarillo, TX). A549 was cultured in RPMI 1640 medium (Cytiva, Utah, USA) with 10% fetal bovine serum. All cells were cultured in 37°C humidified incubator with 5% CO_2_.

### 2.4. RNA Isolation and Real-Time PCR Analysis

According to the manufacturer's protocol, total RNA was isolated from cell lines by using TRIzol reagent (Invitrogen, USA). Complementary DNA (cDNA) was obtained using a PrimeScript RT reagent kit (TaKaRa, Japan). Real-time PCR was performed using TB Green Premix Ex Taq II (TaKaRa, Japan) in a Light Cycler 480 II Real-Time PCR system. Glyceraldehyde phosphate dehydrogenase (GAPDH) was employed as a control for normalization. The primers were shown as follows: FH forward 5′-CCGCTGAAGTAAACCAGGATTATG-3′ and FH reverse 5′-ATCCAGTCTGCCATACCACGAG-3′; and GAPDH forward 5′-GTCTCCTCTGACTTCAACAGCG-3′ and GAPDH reverse 5′-ACCACCCTGTTGCTGTAGCCAA-3.

### 2.5. Correlation between FH Expression Level and Patients' Prognosis

The relationship between FH expression and OS in 33 types of cancer was analyzed by forest plots and the Kaplan-Meier curves. The hazard ratio (HR) and log-rank *P* values were acquired by univariate survival analysis.

### 2.6. Association between FH and Tumor Immunity

The Tumor Immune Estimation Resource (TIMER) database (https://cistrome.shinyapps.io/timer/, v2.0) was used to obtain immune infiltrating cell scores for 33 cancer types. The associations between FH levels and 6 immune infiltrates cells—B cell, CD4^+^ T cell, CD8^+^ T cell, neutrophil cell, macrophage cell, and dendritic cell—were evaluated by the Spearman correlation analysis. Moreover, using the Pearson correlation analysis, we examined the correlation between FH level and immune checkpoint marker level.

### 2.7. Statistical Analysis

The expression level of FH in different tissues was analyzed by *t* test. The univariate survival analysis and Kaplan-Meier survival analysis were used to analyze the correlation between FH expression and patients' overall survival. *P* < 0.05 were considered significant for all statistical analysis.

## 3. Results

### 3.1. Genomic Alterations of FH in Human Pan-Cancer

**Figure 1:**
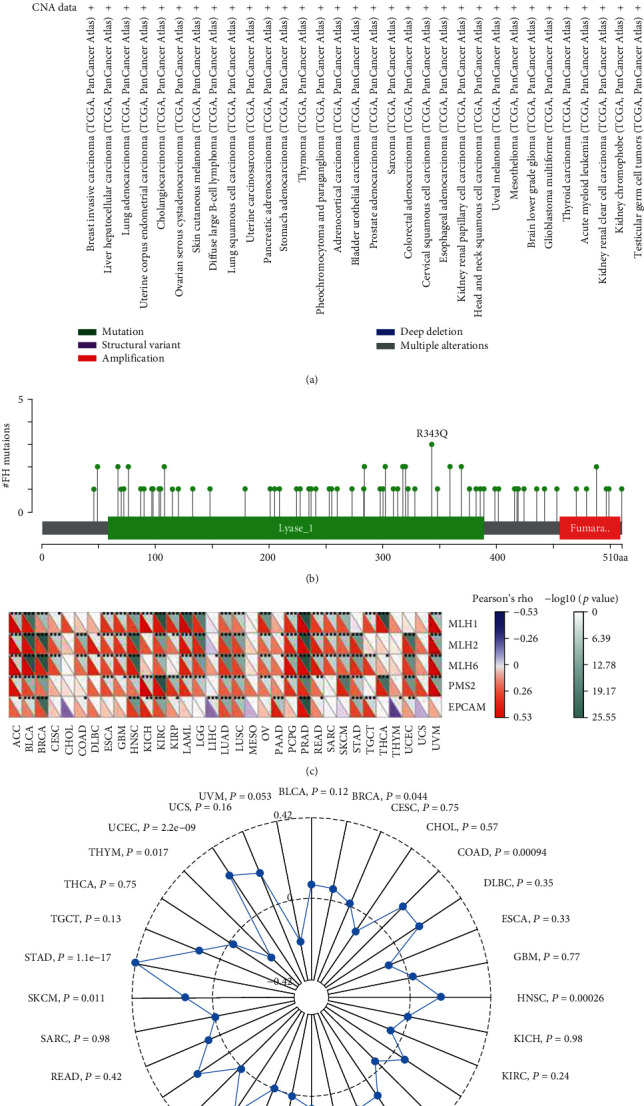
Genomic alterations of FH in human pan-cancer. (a) The alteration frequency of FH in human pan-cancer. (b) The types and distributions of FH mutations. *X*-axis: amino acid; *Y*-axis: numbers of FH mutations; green/red box: RNA recognition motif (190-248aa, 376-444aa, and 471-527aa); #: number of FH mutations. (c) The association between FH expression level and four MMR genes mutation. (d) Radar map showing the correlation between FH expression and TMB. ^∗^*P* < 0.05, ^∗∗^*P* < 0.01, ^∗∗∗^*P* < 0.001.

As we all know, genomic mutation is closely related to tumorigenesis [22]. Using the cBioPortal database, we identified genomic alterations of FH in 32 cancers, including mutations and copy number variations. As a result, FH was mutated or copy number varied in 27 cancers. The results showed that FH mutation frequencies are high in UCEC, BLCA, HNSC, and LAML. Furthermore, FH amplification was one of the significant single factors for alteration in CHOL, USC, PCPG, ESCA, and KIRC (Figure 1(a)). In addition, 73 FH mutations were identified across pan-cancer, and all of them (100%) were missense (Figure 1(b)).

DNA mismatch repair (MMR) maintains genomic stability [23]. Mutations in MMR gene might cause defective mismatch repair, leading to genomic alterations of some genes [24]. Next, we investigated the correlation of four MMR genes' mutation and FH. As shown in Figure 1(c), in most types of cancers, such as LUAD, BLCA, and LUSC, FH expression was significantly related with the mutation level of MMR genes. We next explored the relationship between FH expression and tumor mutational burden (TMB) level. FH expression was associated with TMB in BRCA, COAD, HNSC, LGG, LIHC, LUAD, PAAD, PRAD, SKCM, STAD, THYM, and UCEC (Figure 1(d)). All these results indicate FH shows genomic alterations in many cancers.

### 3.2. The mRNA Expression of FH in Human Pan-Cancer

**Figure 2:**
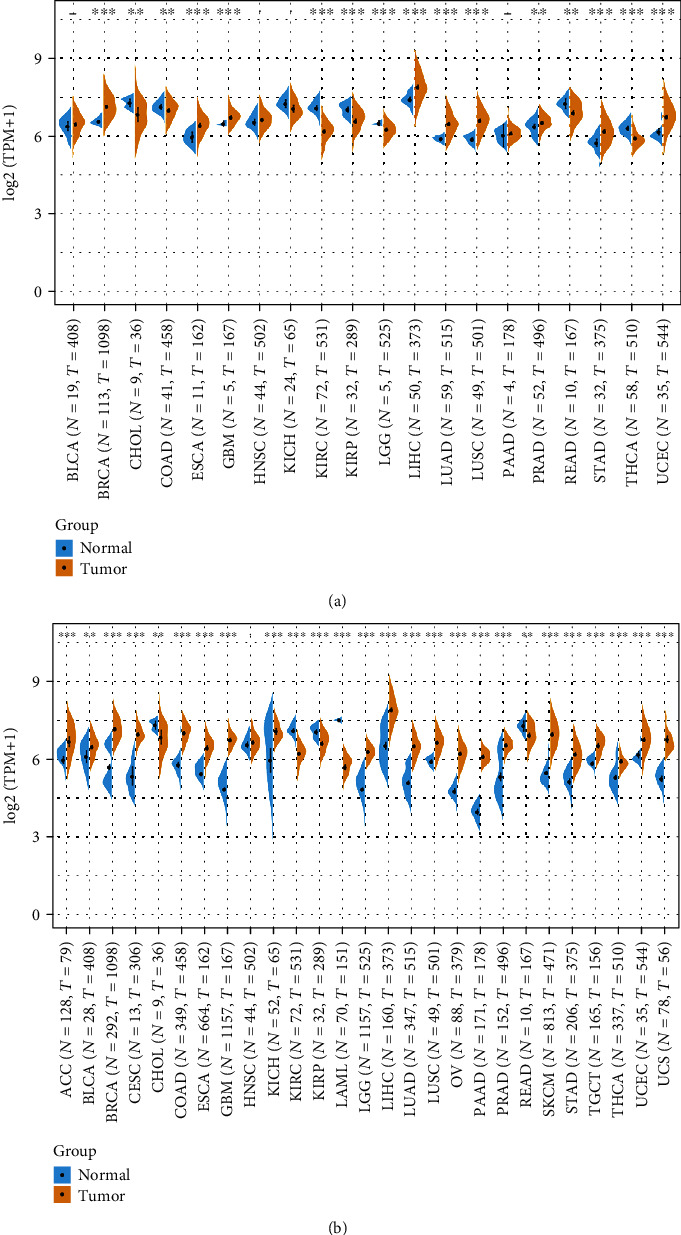
Expression level of FH in different cancer types. (a) Expression level of FH in tumor and normal tissues from TCGA database. (b) Expression level of FH in tumor and normal tissues from TCGA and the GTEx database. ^∗^*P* < 0.05, ^∗∗^*P* < 0.01, ^∗∗∗^*P* < 0.001.

Next, the FH level between tumor tissues and normal tissues in 20 types of cancers was obtained from TCGA database. FH was overexpressed in BRCA, ESCA, GBM, LIHC, LUAD, LUSC, PRAD, STAD, and UCEC tissues compared with normal tissues (Figure 2(a)). In addition, we combined the GTEx database to expand the normal tissue data. Furthermore, the expression level of FH in 27 tumors was analyzed. As shown in Figure 2(b), FH was upregulated in 21 types of cancer tissues, including CC, BLCA, BRCA, CESC, COAD, ESCA, GBM, KICH, LGG, LIHC, LUAD, LUSC, OV, PAAD, PRAD, SKCM, STAD, TGCT, THCA, UCEC, and UCS. These results suggest that FH is abnormally upregulated in various cancers.

### 3.3. Prognostic Value Analysis of FH in Human Pan-Cancer

**Figure 3:**
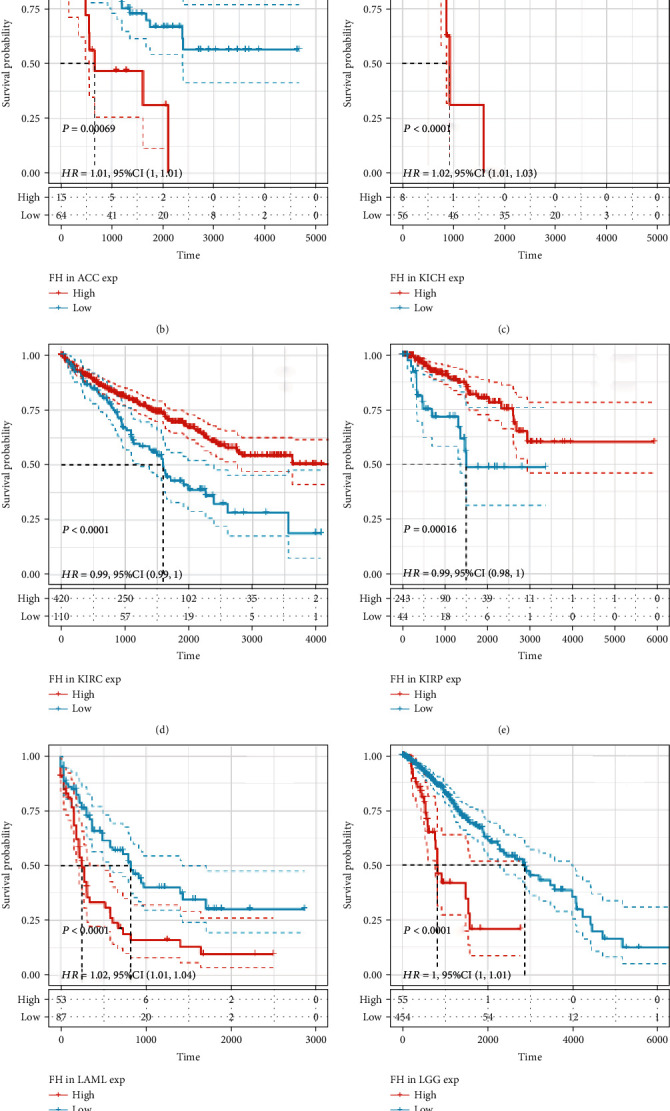
Association between FH expression level and patients' OS. (a) Forest plots of hazard ratios (HRs) of FH expression level in 33 tumor types. (b) The Kaplan-Meier analysis of the correlation between FH expression and patients' OS in ACC, KICH, KIRC, KIRP, LAML, LGG, LUAD, and SKCM.

Next, we investigated whether abnormal expression of FH affects patients' prognosis. By univariate survival analysis, we found that FH expression was associated with patients' OS in 8 cancer types, including ACC, KICH, KIRC, KIRP, LAML, LGG, LUAD, and SKCM (Figure 3(a)). The Kaplan-Meier curves showed that increased FH expression was correlated with poor prognosis in 6 cancer types including ACC (*P* = 0.00069, HR = 1.01), KICH (*P* < 0.0001, HR = 1.02), LAML (*P* < 0.0001, HR = 1.02), LGG (*P* < 0.0001, HR = 1), LUAD (*P* = 0.014, HR = 1), and SKCM (*P* < 0.0001, HR = 1). However, KIRC (*P* < 0.0001, HR = 0.99) and KIRP (*P* = 0.00016, HR = 0.99) were exceptions where FH overexpression indicated a better prognosis (Figure 3(b)).

### 3.4. The Association between FH Expression and Tumor Immunity

**Figure 4:**
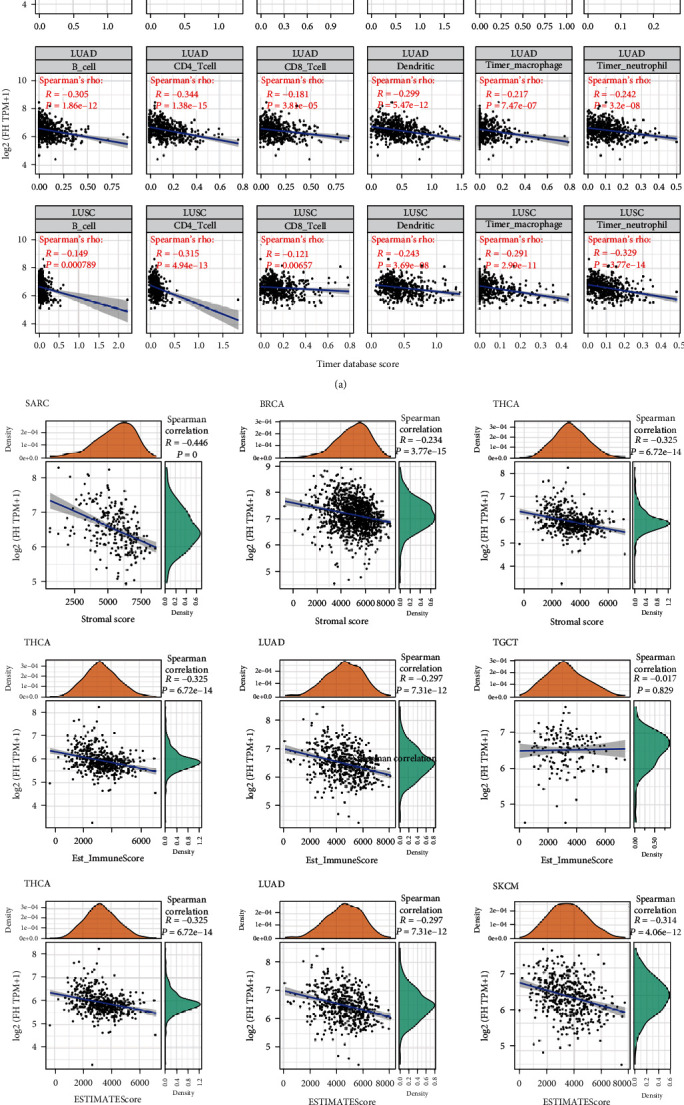
The association between FH expression and tumor immunity. (a) Correlation of FH expression with immune infiltration level of 6 types of immune cells in LIHC, LUAD, and LUSC. (b) Correlation analysis between FH expression and ImmuneScore/Stromal Score/ESTIMATEScore in human pan-cancer.

The immune cells in TME can affect patients' survival [25]. To explore the mechanism of FH affecting patients' prognosis, the correlation between FH expression and immune infiltration in pan-cancer was further investigated. First, we analyzed the scores of 6 types of immune cells (B cell, CD4^+^ T cell, CD8^+^ T cell, neutrophil cell, macrophage cell, and dendritic cell) from 33 cancer types through the TIMER database. Notably, FH level was significantly associated with 6 types of immune cells in LIHC, LUAD, and LUSC (Figure 4(a)). To quantify the immune and matrix components in cancers, the immune score (i.e., ImmuneScore), matrix score (i.e., StromalScore), and estimate score (i.e., ESTIMATEScore) were obtained. FH level was significant negatively associated with the ImmuneScore in SARC, BRCA, THCA, StromalScore in THCA, LUAD, TGCT, and ESTIMATEScore in THCA, LUAD, and SKCM (Figure 4(b)).

**Figure 5:**
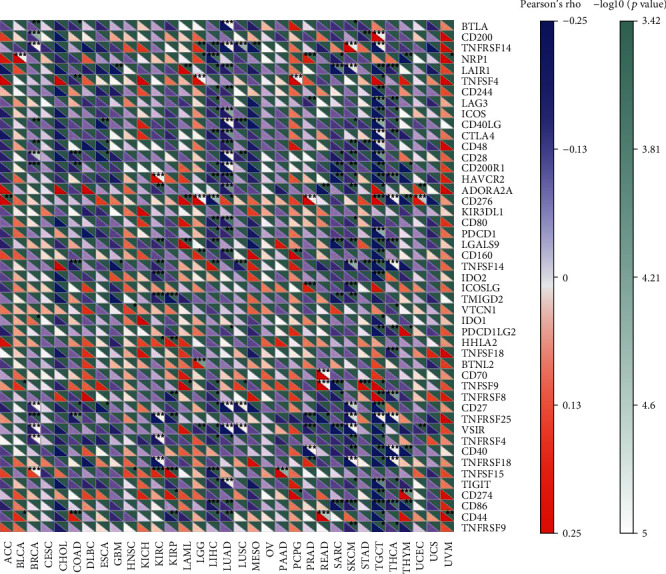
Correlation analysis of FH expression levels with 47 immune checkpoints in human pan-cancer. ^∗^*P* < 0.05, ^∗∗^*P* < 0.01, ^∗∗∗^*P* < 0.001.

Next, we explored how FH affected immune cells infiltration. The correlation between FH expression and immune checkpoint gene expression was investigated. As shown in Figure 5, we found that in some cancers, especially in LUAD and TGCT, FH expression was significantly correlated with multiple immune checkpoint markers, such as BTLA, TNFRSF14, LAIR1, CD48, and CD28.

## 4. Discussion

Pan-cancer analysis can reveal similarities and differences in tumors. In recent years, many studies have used pan-cancer analysis to find biomarkers related to cancer prognosis and immunity [26, 27]. FH protein participates in the tricarboxylic acid (TCA) cycle, where it catalyzes the reversible hydration of fumarate to malate [28]. At present, many studies have shown that TCA is closely related to the occurrence and development of cancer [29, 30]. Therefore, the role of FH in cancer is worth exploring. Non-small cell lung cancer, especially lung adenocarcinoma, is a serious threat to human health and life. It is becoming more and more important to find new treatment methods and targets to improve the prognosis of lung cancer [31, 32]. In this study, we explored the roles of FH in pan-cancer. On the one hand, we investigated genomic alterations of FH in pan-cancer and identified that there were mutations or copy number variations in FH genome. On the other hand, we found FH was upregulated in 21 types of cancers and related to patients' poor prognosis and immunity in LUAD. These results provide new clues for further research on the roles of FH in cancer.

Genomic instability, including genomic mutations and copy number variants, is the major cause of cancer development [33–36]. And research shows MMR gene mutations are closely related to tumorigenesis [37]. Our results showed FH genome mutation or copy number variation in many types of cancers. And FH expression was found significantly related with the mutation level of MMR genes and TMB level. In brief, our results showed that aberrant FH expression might play an important role in tumorigenesis.

FH has been reported to alter cancer cell migratory potential, and hopefully as a therapeutic target in renal cancer [38]. In addition, the inhibition of FH can improve the efficacy of cisplatin-mediated chemotherapy in GC [11]. However, the role of FH in other malignancies remains to be determined. In the present study, we found that FH was upregulated in 21 types of cancers tissues than in normal tissues. To further understand the roles of FH in cancer, we explored the prognostic value of FH in pan-cancer. A high expression level of FH was associated with poor prognosis in several types of cancers, particularly in LUAD. And our experimental results also showed that FH level was significantly upregulated in lung cancer cell lines, including lung adenocarcinoma cell line A549 (Supplementary Figure 1). These results strongly indicated that FH is a significant gene in cancer and may be a potential prognostic marker in patients with LUAD.

Recently, tumor immune microenvironment has received extensive attention, and based on the characteristics of immune cells in the TME, immunotherapy was developed and applied to clinical treatment [39]. Tumor immune microenvironment is a double-edged sword: it can inhibit the development of tumors and can also provide favorable conditions for cancer cells to promote the development of tumors [40]. Tumor-infiltrating immune cells, such as B cells, T cells, neutrophils, macrophages, and dendritic cells, play a significant role in tumor immune microenvironment [41] and can affect the occurrence and development of tumors. For instance, B cells can secret immunoglobulins, promote T cell response, and kill cancer cells to inhibit tumor progression [42]. In advanced ovarian carcinoma, the presence of intratumoral T cells associates with improved survival of patients [43]. Neutrophils can stimulate T cell proliferation [44] to suppress tumor progression. Macrophages and dendritic cells are also closely related to tumor progression [45–47]. Interestingly, in our study, it was found that FH level was significantly negatively correlated with immune infiltrating cells in LUAD, LIHC, and LUSC. Moreover, we have noticed that immune checkpoint therapy is a hot spot in the treatment of cancer. For example, it can help us to define new means to treat pancreatic cancer [48] and has revolutionized lung cancer treatment paradigms [49]. So, we analyzed the correlations between FH level and immune checkpoint markers; the results showed that FH expression was significantly correlated with a variety of immune checkpoint markers in LUAD and TGCT. However, the results lack validation of clinical specimens, which is the limitation of the study. Overall, our results suggested FH is implicated in cancer immunity, particularly in LUAD.

## 5. Conclusion

In conclusion, we performed a pan-cancer analysis of the FH and elucidated the prognostic and immune significance of FH expression in human cancers. Our observations indicated FH may be an immunotherapeutic target and a potential prognostic biomarker, particularly in LUAD. This study provides new insights into the FH in pan-cancer and novel clues for further exploration of the mechanism of FH in cancer.

## Data Availability

The datasets presented in this study can be found in online repositories. The names of the repositories can be found in the article material.

## Supplementary Materials

Supplementary Materials Supplementary Figure 1: Q-PCR analysis of FH expression in normal and lung adenocarcinoma cells.
