# Supplementary material for: Pan-Cancer Analysis Reveals FH as a Potential Prognostic and Immunological Biomarker in Lung Adenocarcinoma
Source: Dis Markers. 2021 Oct 26;2021:8554844. doi: 10.1155/2021/8554844 (PMC8563123; doi:10.1155/2021/8554844)
Supplement: Supplementary Materials — Supplementary Figure 1: Q-PCR analysis of FH expression in normal and lung adenocarcinoma cells. [file 8554844.f1.docx]

**Supplementary Figure 1**


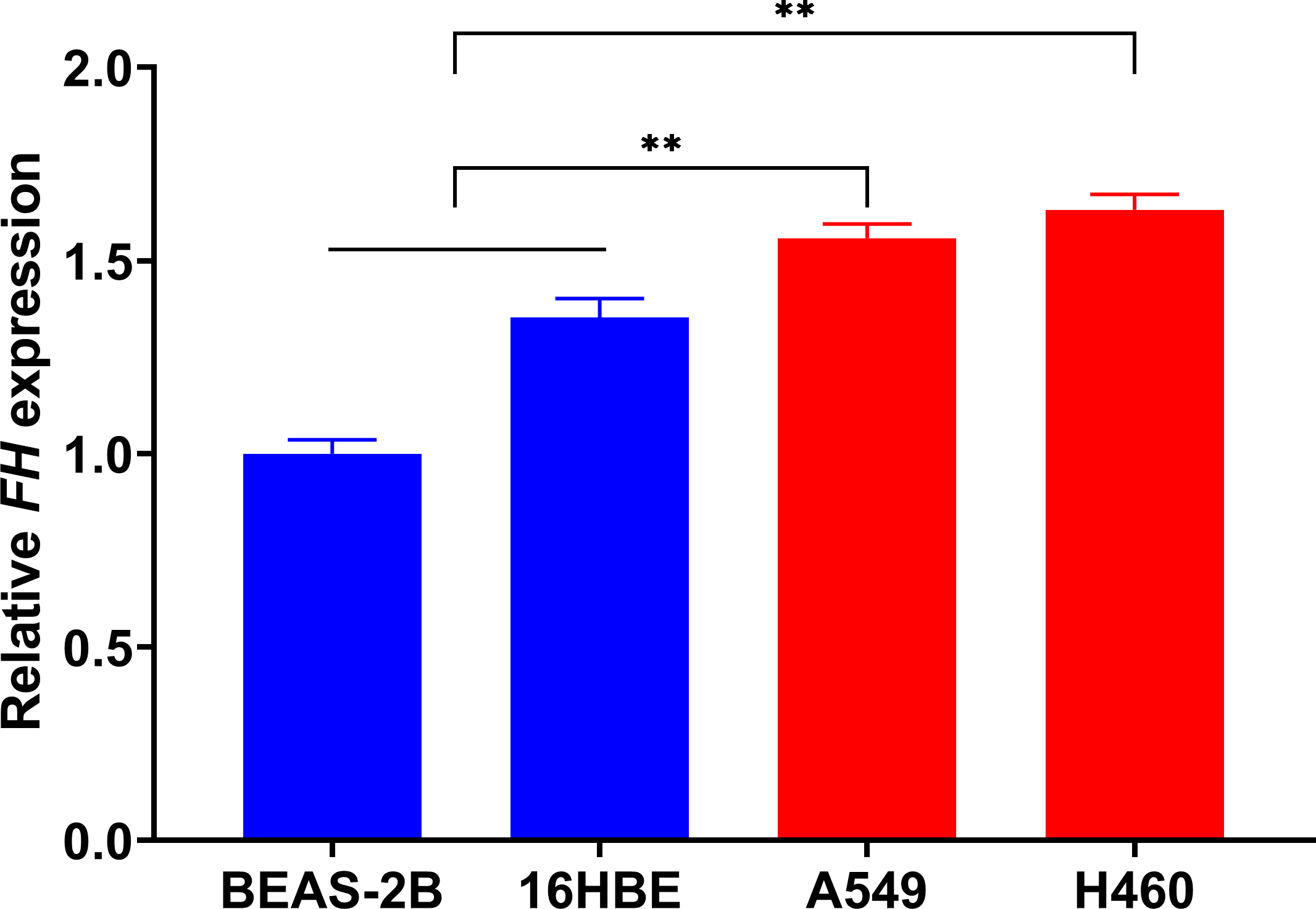


**Supplementary Figure 1.** Q-PCR analysis of *FH* expression in normal and lung adenocarcinoma cells.
